# Conquering Phagophobia: A Journey to Overcoming the Fear of Choking

**DOI:** 10.1155/2024/8827460

**Published:** 2024-08-26

**Authors:** R. Rijal, P. Pokhrel

**Affiliations:** Rhythm Neuropsychiatry Hospital and Research Centre Private Limited, Aloknagar, Kathmandu, Nepal

## Abstract

Phagophobia is a rare and debilitating mental health condition characterized by an intense fear of choking solid food or liquids. Usually there is no underlying anatomical or physiological abnormalities. Choking phobia can lead to the avoidance of solid foods and liquids. This can give rise to other psychiatric disorders like major depressive disorder and anxiety disorder. Only few case reports of choking phobia are available in the literature. Here we present the case of a middle-aged man, with a 10-year history of fear of choking, starting after an aspiration episode and later maintained by a similar episode. The patient felt that food would be stuck in the windpipe, and he could not breathe while swallowing solid food and liquids. This eventually led to reduced eating and drinking causing significant weight loss. He also isolated himself and became depressed. After several searches for help in somatic healthcare, including surgery for deviated nasal septum, the patient was finally investigated in a psychiatric clinic and treated with pharmacological measures and behavioral therapy with considerable improvement within a few months. Choking phobia can mimic different physical conditions and is often misdiagnosed. Early recognition and timely referral to mental health professionals are vital for effective management.

## 1. Introduction

Phagophobia or choking phobia is a rare condition characterized by intense fear of choking. Most studies suggest a prevalence rate between 7% and 9% [1]. In this condition, usually no anatomical or physiological abnormalities can be identified. Sometimes the patients present with other aversive oral experiences like throat infection, dental issues, and repeated vomiting episodes. Patients with this condition can present with actual choking experiences that is often traumatic in nature. Begotka et al. [2] found that out of eight children with phagophobia, the triggering events included actual choking of food (50%), a viral illness (37.5%), or viral illness plus a choking episode (12.5%). On some occasions patients can present with the disorder even without any precipitating factors related to eating or drinking [3]. This is often followed by an intense fear to swallow food that often progresses within hours to days.

This disorder is classified under the “Other (300.29/F40.298)” category in specific phobia disorder of anxiety disorders in the Diagnostic and Statistical Manual of Mental Disorders, fourth edition (DSM-IV) and DSM-5 [4, 5] and under the diagnostic category of avoidant/restrictive food intake disorder as per International Classification of Disease- (ICD-) 11 [6]. There are only some case reports/series and review articles available in the literature related to this condition [7]. There is no report of phagophobia in Nepali scientific literature.

For the management of this condition, different treatment strategies have been described and suggested [8, 9]. Not much is known about the treatment of choking phobia. Several treatment strategies have been used. Most of the previous reports support the use of pharmacological treatment and behavioral therapy. Still, there is a lack of common consensus over the most appropriate approach. Here we report a case of choking phobia in a 41-year-old man who underwent successful treatment with combined pharmacotherapy and behavior therapy.

## 2. Case Presentation

**Figure 1:**
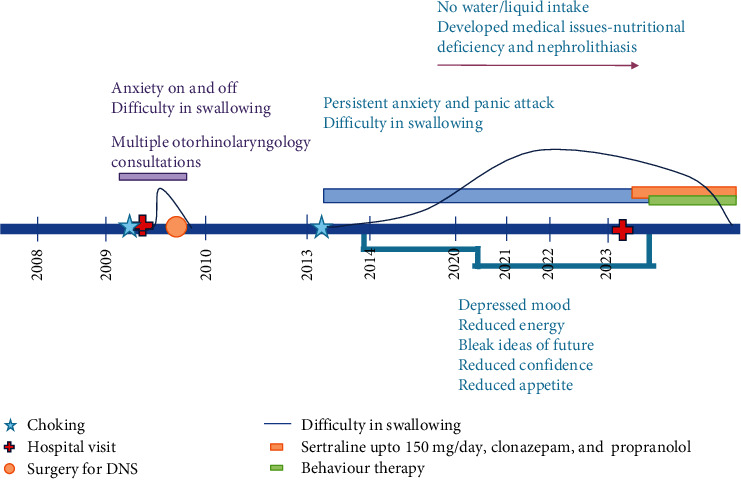
Life chart.

A 41-year-old married man sought consultation in our hospital. He had completed a master's degree in engineering, was an employed resident of Nepal, belonged socioeconomically to the middle class, and had a well-adjusted premorbid personality. He presented with illness of insidious onset (Figure 1).

At the age of 27 years, the patient aspirated while drinking milk. Over days, he started having thoughts of food getting stuck in the throat and inability to breathe. As a result, he began avoiding both more solid and liquid food items. He also often experienced awareness of his own heartbeat and restlessness. Since the symptoms persisted over months, the patient was taken to a private hospital where he was admitted and kept under observation for 4 days, where no cause could be identified for difficulty in swallowing. He continued taking consultations from multiple otorhinolaryngologists. The patient was diagnosed as having deviated nasal septum (DNS) on one of the examinations. It was proposed that the aspiration might be due to this condition, and thus the patient underwent surgery for DNS. After the surgery, the abovementioned symptoms improved over a few days. He sometimes complained of restlessness but was able to eat and drink as before.

The patient was apparently maintaining well till 2013, when he at the age of 31 years drank hot coffee and again aspirated. Following this, within minutes, he felt as if his breathing had stopped and that he was about to die. He started running here and there around the room in his house. The family members got alarmed that something might had happen to him. Within minutes he was taken to a nearby hospital where he again underwent extensive evaluation, but no cause could be found. The patient and family members were reassured, and the patient was told to take some rest. He started breathing normally as before and seemed relaxed during that time. However, following this incident, whenever the patient thought about eating or drinking something or started to eat or drink, he experienced thoughts that food would get stuck, and he could not breathe. He would even have flashbacks related to the incident.

Over a few months, the patient began to avoid social gatherings and eating in public places. His appetite also started to decrease. He stopped drinking water and any liquid food items. It took the patient hours to eat even dry solid food. Whenever he had to eat food, the patient either ate half the portion or went to the washroom and throw away the remaining food. He often felt that he was not able to do what others were doing easily and started to remain sad all day. His family members tried to cheer him up but without success. The patient became more concerned of his health condition and claimed that he would never be fine as before. He often complained that he was feeling weak and made reasons to stay at home. His confidence was further reduced, and he stopped going to any social events. The patient also developed recurrent kidney stones. He was advised to drink plenty of fluid but was unable to do so. By the year 2019, at the age of 37 years, the patient was diagnosed as having vitamin B12 deficiency. He also developed gastritis secondary to *Helicobacter pylori* and received treatment for this condition. During that time, some of his relatives suggested that the patient might be suffering from psychiatric illness as the patient appeared sad, and no cause had been found for his swallowing problem.

The patient visited our hospital in April 2023. He first presented to the outpatient department where in view of his depressive symptoms, he was prescribed sertraline 50 mg/day, clonazepam 0.5 mg/day, and propranolol 20 mg/day. Some improvement in sadness and fatigability was noted.

Two weeks later, a detailed workup was done. The patient was diagnosed with choking phobia and major depressive disorder. During this time, the dose of sertraline was increased to 100 mg/day. Depressive symptoms improved by nearly 60% (as per patient's subjective reporting in follow up), but symptoms of phobia remained. The patient was planned for behavioral therapy. Hierarchy of items causing anxiety and uneasiness were listed down along with subjective unit of distress (SUD) [10]. See Tables 1 and 2 for patient's hierarchy and behavior analysis and self-monitoring work. Jacobson's progressive muscular relaxation exercises (JPMR) [11] was taught, and he was advised to perform these exercises two times a day. Psychoeducation sessions regarding illness and planned therapy were taken with patient and his wife.

Sessions were conducted biweekly in outpatient basis in the hospital. The patient would be made to have food or liquid items starting with the lowest order of the hierarchy. Sometimes videos were recorded during the sessions. These videos were later replayed for the therapist, who would give feedback. During the sessions, the patient's pulse, SUD, and thoughts following the experience were recorded. Exposures were done for 10 min with a break in between and repeated three times for a total of 30 min (Table 3).

The patient was asked to eat the solid or liquid item in the presence of the therapist and to face the anxiety. The patient was asked to do relaxation exercises each time before and after the session. After initial resistance due to the anxiety faced, the patient began to carry out sessions in a structured manner. After three to four sessions, he started facing anxiety and confidence gradually improved. He started carrying out daily sessions at home with wife where she would assist in the exposures.

A total of seven to eight supervised sessions were taken. By the end of 3–4 months, the patient reported very minimal or no anxiety while eating or drinking. He even started going out and interacting with relatives. He would still be hesitant to drink in social events, but the wife noticed significant improvement in terms of quantity and the time taken to eat food or drink liquid items. He also gained around 5–6 kg of weight since the initiation of therapy.

In his final session, in the 1^st^ week of August 2023, the patient was asked to drink water (i.e., the highest task in the hierarchy) in front of the therapist. He would take sips of water and roll it at the back of tongue before finally swallowing it. This observation was made over video recording and was given feedback to normally swallow water. Patient was able to drink water, but it took him 5–7 min to finish a glass of water (previously it would take 20–30 min). Patient started to eat food and liquid items in comparable time as of family members. The patient's dose of clonazepam was tapered and stopped in the last week of August 2023, and sertraline was increased to 150 mg/day.

## 3. Discussion

Choking phobia also known as phagophobia is a distinct illness usually characterized by a stimulus that causes fear of swallowing, which often results in solid or liquid restriction/avoidance [12]. The patients often consult multiple ear, nose,and throat specialists and gastroenterologists before consulting a mental health provider. Even among psychiatrists, choking phobia is commonly misdiagnosed as eating disorder or conversion disorder [9, 12].

In both the episodes, our patient had actual choking experiences followed by difficulty swallowing. For this, he underwent multiple consultations and even surgery for DNS in the 1^st^ episode. Following that, his symptoms reduced, and there was a period in between where the patient maintained apparently well. Again, following actual choking in 2^nd^ episode, he again experienced similar symptoms. This episode caused him to develop depressive illness and impairment in socio-occupational functioning. The patient had to undergo lots of suffering, and the referral to psychiatrist was only undertaken due to his objective depressive symptoms as noticed by his relatives. Thus, this underscores the importance of raising awareness among other medical professionals about choking phobia, associated comorbidity, and need for detailed evaluation of dysphagia [13].

In the literature, two types of choking phobia have been described: post-traumatic type and malingering type. In the first one, the psychic trauma is caused after an experience of gagging or choking. It has been reported that once a patient has actual choking, (s)he will be conditioned to this experience or have flashbacks of similar experiences while trying to swallow something [3, 12]. This condition was similar in our case and led to further avoidance or restriction of food items.

The patient had recurrent panic attacks, which acted as a conditioning factor. Thus, this caused maintenance of long-term anxiety leading to a vicious cycle causing avoidance [3]. This ultimately led to genesis of depressive symptoms, weight loss, and other physical health complications like vitamin deficiency and recurrent kidney stones.

Among the comorbidities secondary to this condition are social anxiety disorder, depressive disorder, and panic disorder [7]. Phagophobia is more common in females (two-thirds of cases) [14], although a recent case series may indicate a more even split between men and women [2]. Phagophobia is highly comorbid with anxiety disorders (15% separation anxiety disorder, 22% obsessive conditions, and 41% panic disorder).

In our case, antidepressant and antianxiety drugs were started before the initiation of nonpharmacological treatment for management of depressive and anxiety symptoms, respectively. Similar management strategy was opted by an Indian study done [13], where the use of antidepressant and a low dose of benzodiazepine was given for depressive symptoms and anxiety related to the choking phobia. This was continued throughout the therapy, and a similar treatment modality was followed in our case as well. As per the treatment protocol available, treatment of phobic disorder consists of psychoeducation, restructuring of cognition, aversion and distraction techniques, and in vivo exposure [15]. We included psychoeducation, behavior analysis, and hierarchy making and tailored it as per the patient's needs. Monitoring of the patient's progress was done through a computerized device (Google Sheet), where both the patient and the therapist had access to the sheet (Tables 2 and 3).

The patient would keep a track of his improvement, and the therapist would give repeatedly feedback. Later, after analyzing the hierarchy, in vivo exposure would be done in front of the therapist as per the ascending hierarchy. In a span of 3–4 months, all these steps led to remission and thus return of the patient to his premorbid self. This is first of its kind study where self-monitoring and hierarchy making were done through the use of Google Sheet.

For someone who was struggling to eat and drink something as basic as water during the last 8–9 years, the patient's motivation and the support from family members helped him to gain confidence to complete sessions and live his life as before.

## 4. Conclusion

This case highlights the use of appropriate treatment strategies for conditions like choking phobia. It also underscores the importance of raising awareness among other medical professionals about choking phobia and the need for a detailed evaluation. However, more trials and studies are needed with regard to its management strategies that can be acceptable throughout the world.

Use of Google Sheet in the psychotherapy can be used even in the treatment of other disorders like obsessive–compulsive disorder and trichotillomania. This is especially true when we live in an era where telepsychiatry is rapidly widening its horizons.

## Figures and Tables

**Table 1:** Sample of exposure session to food (watermelon).

Instruction: Patient was asked to eat 1 slice of watermelon in front of the therapist			
Time	SUD (%)	Pulse (beats/min)	Thoughts

2:00 p.m.	60	88	It might get stuck in throat, and I might not be able to swallow.

Instruction: Patient was asked to eat 2–3 slices of watermelon in front of the therapist			

2:10 p.m.	50	80	I feel uncomfortable, but I was able to swallow.

Instruction: Patient was asked to eat remaining part of the fruit in front of the therapist			

2:20 p.m.	20	80	I thought I could not swallow. But I was able to do it.

The session would end with cognitive restructuring. Attention would be given throughout the session that he would not neutralize his thoughts while swallowing.

**Table 2:** Sample of hierarchy making.

Type of food/liquid	Anxiety noted/SUD (0%–100%)	Thoughts following
Water	100	I might not be able to swallow
Black tea	80–95	I might not be able to swallow
Milk tea	75–90	I might not be able to swallow
Pineapple	90	I might not be able to swallow
Watermelon	80	I might not be able to swallow
Curd	30	I might not be able to swallow
Mango	20–30	I might not be able to swallow
Apple	20	I might not be able to swallow
Rice + lentils	20	I might not be able to swallow
Banana	15	I might not be able to swallow
Dry fruits	10	—

**Table 3:** Sample of self-monitoring and behavior analysis through Google Sheet.

Date	Time	Food/drink	Physical state	Emotional state	Thoughts preceding	Thoughts following	Situation	Duration
1/8/23	8:00 a.m.	Water	Tiered	Worries	I might not be able to swallow	I might die	At home	30 min
1/8/23	12:00 p.m.	Rice with lentils	As usual	—	I might not be able to swallow. I might have choking again	—	At office	—
1/8/23	4:00 p.m.	Apple, watermelon, and black tea	Tiered	Worries	I might have difficulty swallowing	I might die	At office	5–10 min
1/8/23	8:00 p.m.	Dry snacks	Tiered	Relaxed	I might have choking.	—	At home	—

## Data Availability

All data underlying the results are available as part of the article, and no additional source data are required.
